# Early changes in the circulating T cells are associated with clinical outcomes after PD-L1 blockade by durvalumab in advanced NSCLC patients

**DOI:** 10.1007/s00262-020-02833-z

**Published:** 2021-01-09

**Authors:** Elliot Naidus, Jerome Bouquet, David Y. Oh, Timothy J. Looney, Hai Yang, Lawrence Fong, Nathan E. Standifer, Li Zhang

**Affiliations:** 1 Department of Medicine, University of California San Francisco, San Francisco, CA USA; 2 Department of Medicine, Mills-Peninsula Medical Center, Burlingame, CA USA; 3 Integrated Bioanalysis, Clinical Pharmacology and Safety Sciences, R&D, AstraZeneca, South San Francisco, CA USA; 4 Helen Diller Family Comprehensive Cancer Center, University of California San Francisco, San Francisco, CA USA; 5 Thermo Fisher Scientific, Austin, TX USA; 6 Department of Epidemiology Biostatistics, University of California San Francisco, 550 16th Street, Room 6708, UCSF, Box 3211, San Francisco, CA 94158 USA

**Keywords:** Circulating T cells, Diversity, TCR, PD-L1, NSCLC, Network analysis

## Abstract

Immune checkpoint inhibitors (ICI) are designed to activate exhausted tumor-reactive T cells thereby leading to tumor regression. Durvalumab, an ICI that binds to the programmed death ligand-1 (PD-L1) molecule, is approved as a consolidation therapy for treatment of patients with stage III, unresectable, non-small cell lung cancer (NSCLC). Immunophenotypic analysis of circulating immune cells revealed increases in circulating proliferating CD4 + and CD8 + T cells earlier after durvalumab treatment. To examine durvalumab’s mechanism of action and identify potential predictive biomarkers, we assessed the circulating T cells phenotypes and TCR genes of 71 NSCLC patients receiving durvalumab enrolled in a Phase I trial (NCT01693562, September 14, 2012). Next-generation sequencing of TCR repertoire was performed on these NSCLC patients’ peripheral blood samples at baseline and day 15. Though patients’ TCR repertoire diversity showed mixed responses to the treatment, patients exhibiting increased diversity on day 15 attained significantly longer overall survival (OS) (median OS was not reached vs 17.2 months for those with decreased diversity, *p* = 0.015). We applied network analysis to assess convergent T cell clonotypes indicative of an antigen-driven immune response. Patients with larger TCR clusters had improved OS (median OS was not reached vs 13.1 months for patients with smaller TCR clusters, *p* = 0.013). Early TCR repertoire diversification after durvalumab therapy for NSCLC may be predictive of increased survival and provides a mechanistic basis for durvalumab pharmacodynamic activity.

**Supplementary Information:**

The online version contains supplementary material available at 10.1007/s00262-020-02833-z.

## Introduction

The approval of anti-PD-1 and anti-PD-L1 immune checkpoint inhibitors (ICIs) represents a key innovation in the treatment of NSCLC. ICI therapy extends survival in stage III–IV NSCLC [1–4]. However, objective response rates to ICIs remain low at 15–30% [5].

Increased expression of PD-L1 by tumor cells augments response to immunotherapy as well as survival [5]. However, only a minority of patients have tumors expressing high PD-L1 [6]. The successes and shortcomings of ICI therapy in stage III–IV NSCLC have motivated research into alternate immune checkpoint targets and combination ICI therapies [7]. Responses to anti-PD-1/PD-L1 antibodies occur even with low PD-L1 tumor expression [2], yet little is known about the immunologic determinants of such responses.

There is a need to identify clinical variables and biomarkers predictive of response to ICI therapy. Proliferation of peripheral blood Ki67 + PD-1 + CD8 + T cells [8, 9], presence of CD8 + T cells at the tumor margin and high tumor PD-L1 expression [10] correlate with better response to ICIs. The T cell receptor (TCR) repertoire represents the spectrum of TCR antigen specificities that the body can recognize. The TCR repertoire is a potential attractive biomarker for evaluating responses to checkpoint blockade because ICI therapy depends on T cell antigen recognition.

Static and dynamic measurements of peripheral T cell and tumor-infiltrating lymphocyte (TIL) TCR repertoires have shown varying results as a predictive biomarker in immuno-oncology. Increased repertoire diversity should theoretically increase the likelihood of a T lymphocyte recognizing a tumor-specific antigen. In NSCLC patients, increased peripheral blood TCR diversity after anti-PD-1 treatment and high overlap between pre- and post-treatment TCR repertoires have been shown to improve survival [11, 12]. However, others have shown that increased clonality (i.e., decreased diversity*)* of peripheral PD-1 + CD8 + T cells after ICI therapy correlates with longer progression free survival [13]. In melanoma, low baseline TCR repertoire diversity is correlated with improved survival after combination anti-PD-1 plus anti-CTLA-4 treatment [14], yet in pancreatic ductal adenocarcinoma low baseline TCR diversity correlates with improved survival after anti-PD-1 therapy but worse survival after anti-CTLA-4 therapy [15]. In addition, in a study of 24 different solid tumors types treated with anti-PD-1 or anti-PD-L1 therapy, peripheral TCR-ß chain diversity increased in patients that demonstrated partial responses relative to those with progressive or stable disease [16].

These discordant data highlight that TCR repertoire metrics may be associated with different outcomes depending on the type of malignancy, immune perturbation (PD-1/PD-L1 or CTLA-4 blockade), and compartment assayed (peripheral T cells vs TILs).

In the current study, we evaluated peripheral blood TCR-ß chain repertoires in advanced NSCLC before and after treatment with durvalumab (anti-PD-L1) to identify how TCR repertoires are associated with outcome.

## Materials and methods

### Study schema

Blood samples evaluated in this study were collected as part of a Phase 1/2 evaluating durvalumab in patients with advanced solid tumors (NCT01693562). Subjects received durvalumab as either first-line or subsequent therapy. Patients received durvalumab 10 mg/kg every 2 weeks for 12 months or until confirmed progressive disease or unacceptable toxicity. The study was conducted in accordance with the principles of the Declaration of Helsinki, the International Conference on Harmonisation Good Clinical Practice guidelines, and local regulatory requirements. The study protocol was reviewed and approved by the Institutional Review Boards or Ethics Committees of the participating centers, and informed consent was obtained. Biological samples and clinical data were collected at three time points: screening, pre-infusion (cycle 1, day 1; C1D1), and before dose 2nd infusion (cycle 1, day 15; C1D15).

### TCRB library preparation, sequencing, and clonotyping

Peripheral blood mononuclear cells (PBMC) were isolated from 12 mL of blood. DNA was extracted from approximately 0.5 million cryopreserved PBMC per sample via the QIAGEN AllPrep Kit (Qiagen), followed by quantitation via the Invitrogen Qubit dsDNA HS assay (Thermo Fisher Scientific). A target of 100 ng gDNA was used as input for library preparation via the Oncomine TCRB-SR DNA assay. Libraries were sequenced via the Ion Gene Studio S5 using the 540 chip (Thermo Fisher Scientific) to a target depth of 2 million reads per library. Clonotyping and reporting of secondary repertoire features was performed via Ion Reporter 5.10.

### TCR data assessment

Only samples with unique clones ≥ 1000, read depth ≥ 800,000 and ≥ 40% productive reads were retained for TCR data analysis. Diversity of the TCR repertoire at each time point was measured using *clonality* on a scale of 0 to 1, indicating that all clonotypes are equally common or the TCR repertoire is dominated by a single clone, respectively [17]. TCR convergence frequency (TCF) was calculated as the aggregate frequency of clones sharing an amino acid sequence with at least one other clone [18]. TCR repertoire change from baseline to 14 days after treatment was evaluated by relative clonality (RCL, i.e., ratio of clonality after durvalumab relative to baseline,) and relative TCF (RTCF) which is defined as in the same fashion.

### Network analysis

As in [19], for each patient a pairwise distance matrix of each pair of amino acid sequences was calculated based on Levenshtein distance. A convergent group was defined as the cluster that included the clones with the distance ≤ to 1 (allowing maximum of 1 bp difference among amino acid sequences). Network visualization was performed using R packages: ape and igraph. The diameter, the largest number of vertices which must be traversed to travel between two vertices, was used to describe the property of the network for each sample, and is calculated using a breadth-first search-like method [20].

### Statistical analysis

Frequency and percentage were used to summarize categorical variables, and median with range was used to summarize continuous variables. Two group comparisons were performed using Wilcoxon rank-sum test for continuous variables and Pearson’s Chi-squared test for categorical variables. Overall survival (OS), defined as the time from the start of treatment to the date of death due to any cause, was estimated by the Kaplan–Meier method. The relationship between OS and TCR features as well as other covariates was analyzed using a log-rank test and Cox proportional hazards (CPH) models. The multivariable CPH model was built using backward stepwise selection on the full model with every variable, retaining variables with *p* < 0.2 in the final model. Statistical significance was declared at *p* < 0.05, and no multiple testing adjustment was done. All statistical analysis was done with the software R (https://www.r-project.org/).

## Results

A total of 74 NSCLC patients had PBMCs available for next-generation TCR sequencing. We took C1D1 as the baseline to ensure a consistent interval of 14 days to assess changes in peripheral TCR repertoire after durvalumab exposure. We retained 71 patients with high-quality TCR sequencing data at either time point for the TCR-related analysis, of which 62 patients had samples from C1D1, 61 from C1D15, and 52 from both time points.

### Baseline characteristics

These 71 patients incorporated roughly equal numbers of patients along the lines of sex, tumor histology, and age greater than 65 years (Table 1). Around 90% of patients were current or prior smokers, similar to published ICI trials in NSCLC [1–3]. Durvalumab was used as 1st line therapy in 31% of patients and 2nd or higher line therapy after progression in the remainder. Staining for PD-L1 was low or negative in 29% of patients. After receiving durvalumab, 31% of patients had progressive disease at initial follow up CT scan while 47.3% had stable disease and 21.6% had a radiographic clinical response, similar to prior trial data [4].

**Table 1:** Baseline (prior to durvalumab therapy) clinical and demographic characteristics of the 71 patients who have high-quality TCR sequencing data in either time points

Characteristic	Number of patients (*N* = 71)
Male sex—no. (%)	39 (54.9)
Age ≥ 65 years—no. (%)	36 (50.7)
Squamous histology—no. (%)	37 (52.1)
Caucasian race—no. (%)	71 (100)
PD-L1 low/negative—no. (%)	20 (29.0)
ECOG score of 1 at baseline—no. (%)	45 (63.4)
Smoking history—no. (%)	
Current	13 (18.3)
Former	53 (74.6)
Never	5 (7.0)
Prior line of therapy—no. (%)	21 (29.6)
Liver metastases at baseline—no. (%)	19 (27.5)
Response	
Progressive disease	21 (29.6)
Stable disease	34 (47.9)
Response	16 (22.5)

### TCR repertoire dynamics after durvalumab predict overall survival

**Fig. 1:**
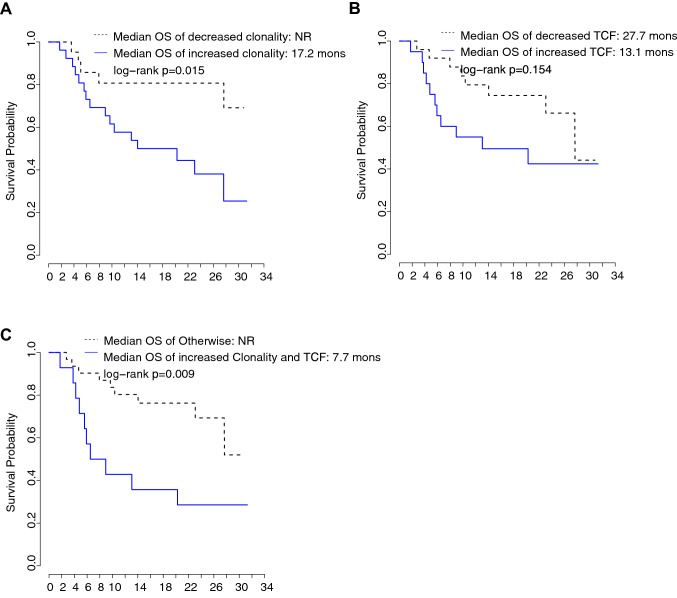
The relationship of TCR repertoire changes with overall survival (OS). **a** Kaplan–Meier curves of OS for the patients with increased (solid line) vs decreased (dashed line) TCR repertoire clonality from baseline to post-treatment. **b** Kaplan–Meier curves of OS for the patients with increased (solid line) vs decreased (dash line) TCR convergence frequency (TCF) from baseline to post-treatment. **c** Kaplan–Meier curves of OS for patients with increased clonality (CL) and increased TCF (solid line) from baseline to post-treatment vs rest of the patients (dashed line)

While baseline clonality was not correlated with OS (Table 2), an increase in RCL (i.e., decreased diversity) was associated with decreased OS (hazard ratio (HR) = 2.37 with 95% confidence interval (CI) [1.28, 4.38], *p* = 0.006). Median OS for patients with decreased clonality after treatment (RCL < 1) was not reached (NR) versus 17.2 months for patients with increased clonality (RCL ≥ 1) (Fig. 1a). Supplementary Table 1 presents individual clonality and survival metrics for all patients. After accounting for patient demographic and clinical characteristics, the multivariable CPH model showed that increased clonality showed a trend toward decreased OS (HR = 2.79 with 95% CI [0.90, 8.66], *p* = 0.075), though not statistically significant.

**Table 2:** Univariable and multivariable Cox proportional hazards modeling of clinical variables and relative clonality change from baseline to post-treatment with overall survival

	Univariable analysis			Multivariable analysis		
	HR^a^	95% CI^b^	*p*	HR^a^	95% CI^b^	*p*
Male sex	1.56	(0.79, 3.08)	0.197	2.14	(0.79,5.75)	0.134
Age ≥ 65 years	1.71	(0.87, 3.34)	0.119	2.06	(0.76,5.55)	0.154
Squamous histology	1.68	(0.83, 3.39)	0.147			
PD-L1 low/negative	2.01	(0.99, 4.10)	0.054	1.94	(0.74,5.11)	0.178
ECOG score of 1 at baseline	2.34	(1.09, 4.99)	0.028			
Ever-smoker	0.44	(0.17, 1.15)	0.093	2.65	(0.66,10.63)	0.169
Prior line of therapy	2.09	(0.91,4.80)	0.081	0.45	(0.16,1.25)	0.124
Liver metastases at baseline	1.94	(0.95, 3.99)	0.070			
Baseline clonality	6.21	(0.25,156.03)	0.267			
Increased clonality^c^	3.32	(1.20, 9.20)	0.021	2.79	(0.90,8.66)	0.075

^a^HR: hazard ratio

^b^95% CI: 95% confidence interval

^c^Increased clonality: defined based on clonality at day 15 is greater than baseline clonality

In multivariable analysis, male sex (*p* = 0.045) and the presence of liver metastases (*p* = 0.044) were associated with increasing repertoire clonality after durvalumab suggesting they might be the confounders in the relationship between RCL and OS.

### TCR convergence frequency adds predictive value beyond repertoire clonality

TCR convergence refers to the development of similar TCR antigen specificity despite different amino acid or nucleotide sequences for the TCR-ß chain. We found a trend toward increased OS among patients with a decrease in TCF after treatment (median OS 27.7 and 13.1 months for RTCF < 1 and ≥ 1, respectively, log-rank test *p* = 0.154) (Fig. 1b). Patients with either decreased clonality or decreased TCF after treatment had significantly increased median OS (NR *vs* 7.7 months, log-rank test *p* = 0.009) (Fig. 1c). However, in multivariable analysis, this finding was no longer statistically significant (HR = 2.76, 95% CI [0.76, 10.03], *p* = 0.124).

### Increased complexity of the TCR repertoire network is associated with longer survival

**Fig. 2:**
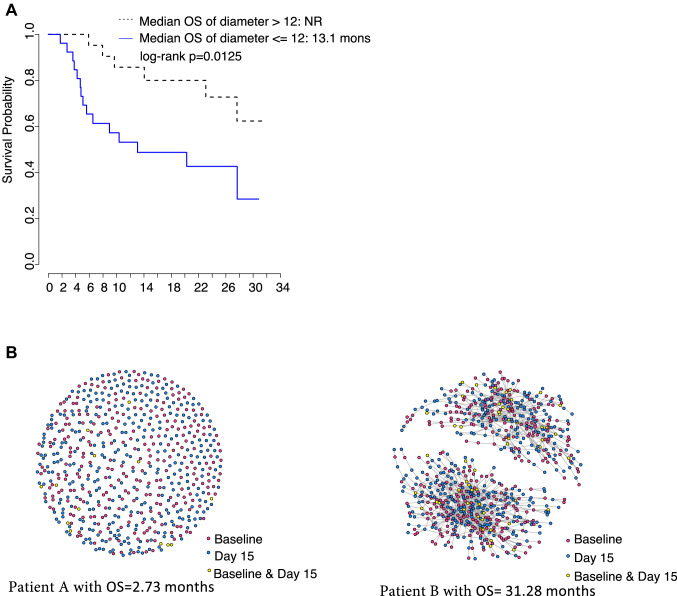
The relationship of TCR network properties with overall survival (OS). **a** Kaplan–Meier curves of OS for the patients with larger diameters (dashed line) vs shorter diameters (solid line). **b** Network figures for the two representative patients. Patient A with OS of 2.7 months, while Patient B with OS of 31.3 months. Each node represents a single amino acid sequence colored by the time points. The nodes connected by lines were within the same convergent group

We applied network analysis of each patient’s TCR repertoire based on the similarity of amino acid sequences. To obtain inferences across patients, we correlated network diameter with OS. TCR networks with larger diameters correlate with increased OS (HR = 0.31, 95% CI [0.12, 0.83], *p* = 0.018). Median OS was NR vs 13.1 months for patients with higher and lower diameter networks, respectively (diameter > 12 vs ≤ 12, where 12 was the median diameter) (Fig. 2a). Two representative patient TCR network diagrams are shown for illustration (Fig. 2b). After adjusting for baseline liver metastases and gender, HR was 0.35 (95% CI [0.12, 1.02], *p* = 0.055).

## Discussion

TCR repertoire diversity was similar from baseline to 14 days post-treatment in durvalumab-treated NSCLC patients, though an increasing TCR repertoire clonality (decreased diversity) was associated with shorter OS. Interestingly, patients with both increasing clonality and TCF (dually increased group) had even worse survival. This indicates that peripheral T cells clones that expanded or converged upon specific antigens were ineffective in controlling tumor growth or potentially caused harm if directed toward self-antigens. TCF adds supplemental predictive value to repertoire clonality for predicting clinical outcomes [18], but TCF was increased in patients who had a response to anti-CTLA-4 monotherapy in contrast to the increased TCF correlating with shorter survival as we describe with durvalumab. Anti-PD-L1 and anti-CTLA-4 blockade likely have different immunologic effects and others have found that baseline TCR clonality can produce differential outcomes with each therapy in the context of pancreatic ductal adenocarcinoma [15], so the finding that increasing TCF is associated with worse survival after anti-PD-L1 blockade but improved survival after anti-CTLA-4 blockade may not be contradictory. If CTLA-4 inhibits T cell priming while PD-1/PD-L1 inhibits T cell effector function, one possible explanation may be that a narrowing of the immune response with PD-L1 blockade may reflect non-productive antigen recognition of already exhausted T cells, while repertoire narrowing following CTLA-4 blockade may represent generation of novel, productive T cell responses to a limited number of tumor antigens.

Our application of network analysis to the TCR repertoire showed that more complicated TCR networks were associated with longer survival, indicating an antigen-driven immune response. This method complements standard metrics of TCR diversity, but yielded the concordant finding that peripheral TCR repertoires, which become more diverse with more complex networks and less TCR convergence are associated with improved OS.

The loss of statistical significance in the multivariable model might be due to both the small sample size relative to model variables and unidentified confounders influencing survival independently of immune-specific mechanisms. Larger stratified randomized trials might overcome these issues where potential confounding covariates could serve as stratification factors. However, as discussed in recent publications and statistical forums, our focus should not be restricted by *p* < 0.05, the actual effect size would be more important.

In summary, we found that early TCR repertoire diversification may be predictive of increased survival and provides a mechanistic basis for durvalumab pharmacodynamic activity. Future work must clarify if increased OS associated with increasing TCR diversity is due to proliferation of many rare anti-tumor clones or elimination of common clones that are ineffective at tumor control.

### Supplementary Information

Below is the link to the electronic supplementary material.

Supplementary file1 (DOCX 26 KB)

## Data Availability

Data underlying the findings described in this manuscript may be obtained in accordance with AstraZeneca’s data sharing policy described at https://astrazenecagrouptrials.pharmacm.com/ST/Submission/Disclosure

## Code availability

A publicly available R software “TCR3D” is available at https://github.com/mlizhangx/TCR-3D.
