# Supplementary material for: Early changes in the circulating T cells are associated with clinical outcomes after PD-L1 blockade by durvalumab in advanced NSCLC patients
Source: Cancer Immunol Immunother. 2021 Jan 9;70(7):2095–102. doi: 10.1007/s00262-020-02833-z (PMC8195930; doi:10.1007/s00262-020-02833-z)
Supplement: Supplementary file 1 — Supplementary file1 (DOCX 26 KB) [file 262_2020_2833_MOESM1_ESM.docx]

Supplementary Table 1 – Individual clonality and survival metrics for all patients, ordered by relative clonality (RCL).

| Patient ID | Clonality Baseline | Clonality  Day 15 | Relative Clonality | OS (month) | Alive (0)  /Dead (1) |
| --- | --- | --- | --- | --- | --- |
| 6 | 0.195 | 0.048 | 0.244 | 30.066 | 0 |
| 41 | 0.209 | 0.113 | 0.538 | 20.066 | 0 |
| 37 | 0.421 | 0.268 | 0.635 | NA | NA |
| 52 | 0.044 | 0.028 | 0.650 | 4.671 | 1 |
| 2 | 0.094 | 0.062 | 0.656 | 27.664 | 1 |
| 59 | 0.089 | 0.060 | 0.674 | 12.467 | 0 |
| 43 | 0.075 | 0.053 | 0.708 | 7.928 | 1 |
| 32 | 0.474 | 0.344 | 0.727 | 3.586 | 1 |
| 68 | 0.045 | 0.033 | 0.744 | 12.697 | 0 |
| 69 | 0.053 | 0.040 | 0.750 | 21.908 | 0 |
| 12 | 0.054 | 0.041 | 0.765 | 5.066 | 1 |
| 60 | 0.105 | 0.086 | 0.817 | 16.020 | 0 |
| 28 | 0.058 | 0.048 | 0.827 | 5.757 | 0 |
| 21 | 0.040 | 0.033 | 0.831 | 29.243 | 0 |
| 74 | 0.057 | 0.048 | 0.832 | 12.434 | 0 |
| 26 | 0.287 | 0.240 | 0.836 | 30.395 | 0 |
| 54 | 0.053 | 0.045 | 0.842 | 20.263 | 0 |
| 8 | 0.096 | 0.083 | 0.866 | 30.757 | 0 |
| 34 | 0.556 | 0.484 | 0.870 | NA | NA |
| 11 | 0.317 | 0.276 | 0.871 | 30.789 | 0 |
| 29 | 0.086 | 0.075 | 0.872 | 25.066 | 0 |
| 55 | 0.050 | 0.044 | 0.876 | 12.237 | 0 |
| 15 | 0.112 | 0.099 | 0.887 | 30.164 | 0 |
| 13 | 0.032 | 0.029 | 0.905 | 1.414 | 0 |
| 18 | 0.075 | 0.075 | 0.994 | 1.809 | 0 |
| 9 | 0.326 | 0.327 | 1.005 | 27.632 | 1 |
| 19 | 0.061 | 0.061 | 1.005 | 6.513 | 1 |
| 5 | 0.087 | 0.089 | 1.028 | 29.967 | 0 |
| 35 | 0.032 | 0.033 | 1.034 | 18.191 | 0 |
| 73 | 0.317 | 0.327 | 1.034 | 10.362 | 1 |
| 65 | 0.042 | 0.044 | 1.051 | 5.592 | 1 |
| 50 | 0.042 | 0.044 | 1.057 | 16.678 | 0 |
| 45 | 0.290 | 0.311 | 1.072 | 16.349 | 0 |
| 44 | 0.247 | 0.265 | 1.074 | 23.059 | 1 |
| 38 | 0.095 | 0.105 | 1.111 | 23.454 | 0 |
| 31 | 0.144 | 0.162 | 1.127 | 25.164 | 0 |
| 46 | 0.054 | 0.062 | 1.137 | 24.145 | 0 |
| 42 | 0.215 | 0.252 | 1.174 | 3.783 | 1 |
| 23 | 0.045 | 0.054 | 1.201 | 5.888 | 1 |
| 30 | 0.078 | 0.094 | 1.201 | 20.263 | 1 |
| 7 | 0.060 | 0.074 | 1.237 | 31.283 | 0 |
| 22 | 0.094 | 0.122 | 1.293 | 4.770 | 1 |
| 57 | 0.124 | 0.161 | 1.294 | 15.132 | 0 |
| 48 | 0.191 | 0.258 | 1.350 | 1.743 | 1 |
| 16 | 0.039 | 0.053 | 1.359 | 9.671 | 1 |
| 71 | 0.100 | 0.137 | 1.375 | 22.796 | 0 |
| 70 | 0.087 | 0.120 | 1.388 | 14.046 | 1 |
| 17 | 0.147 | 0.222 | 1.509 | 4.211 | 1 |
| 4 | 0.062 | 0.122 | 1.957 | 8.947 | 1 |
| 24 | 0.038 | 0.076 | 1.980 | 1.414 | 0 |
| 1 | 0.031 | 0.081 | 2.578 | 2.730 | 1 |
| 72 | 0.036 | 0.147 | 4.099 | 13.059 | 1 |
| 3 | NA | 0.195 | NA | 2.138 | 1 |
| 10 | 0.057 | NA | NA | 5.197 | 1 |
| 14 | NA | NA | NA | 6.020 | 0 |
| 20 | 0.422 | NA | NA | 2.138 | 1 |
| 25 | 0.205 | NA | NA | 2.270 | 1 |
| 27 | NA | 0.107 | NA | 7.796 | 1 |
| 33 | NA | 0.318 | NA | 19.868 | 0 |
| 36 | NA | NA | NA | NA | NA |
| 39 | NA | 0.038 | NA | 1.711 | 1 |
| 40 | NA | 0.176 | NA | 11.316 | 1 |
| 47 | 0.059 | NA | NA | 15.329 | 0 |
| 49 | 0.094 | NA | NA | 4.737 | 1 |
| 51 | 0.041 | NA | NA | 13.586 | 1 |
| 53 | 0.203 | NA | NA | 9.309 | 1 |
| 56 | NA | 0.111 | NA | 6.118 | 0 |
| 58 | NA | 0.102 | NA | 16.020 | 0 |
| 61 | NA | 0.045 | NA | 19.112 | 1 |
| 62 | 0.461 | NA | NA | 10.263 | 1 |
| 63 | 0.197 | NA | NA | 15.724 | 0 |
| 64 | NA | 0.042 | NA | 5.033 | 0 |
| 66 | 0.059 | NA | NA | 3.355 | 1 |
| 67 | NA | NA | NA | 2.928 | 1 |
